# Predictive performance of aldosterone-to-renin ratio in the diagnosis of primary aldosteronism in patients with resistant hypertension

**DOI:** 10.3389/fendo.2023.1145186

**Published:** 2023-05-08

**Authors:** Fabio Bioletto, Chiara Lopez, Martina Bollati, Stefano Arata, Matteo Procopio, Federico Ponzetto, Guglielmo Beccuti, Giulio Mengozzi, Ezio Ghigo, Mauro Maccario, Mirko Parasiliti-Caprino

**Affiliations:** ^1^ Division of Endocrinology, Diabetes and Metabolism, Department of Medical Sciences, University of Turin, Turin, Italy; ^2^ Clinical Biochemistry Laboratory, City of Health and Science University Hospital, Turin, Italy

**Keywords:** aldosterone-to-renin ratio, aldosterone, renin, primary aldosteronism, resistant hypertension

## Abstract

**Background:**

The systematic use of confirmatory tests in the diagnosis of primary aldosteronism (PA) increases costs, risks and complexity to the diagnostic work-up. In light of this, some authors proposed aldosterone-to-renin (ARR) cut-offs and/or integrated flow-charts to avoid this step. Patients with resistant hypertension (RH), however, are characterized by a dysregulated renin-angiotensin-aldosterone system, even in the absence of PA. Thus, it is unclear whether these strategies might be applied with the same diagnostic reliability in the setting of RH.

**Methods:**

We enrolled 129 consecutive patients diagnosed with RH and no other causes of secondary hypertension. All patients underwent full biochemical assessment for PA, encompassing both basal measurements and a saline infusion test.

**Results:**

34/129 patients (26.4%) were diagnosed with PA. ARR alone provided a moderate-to-high accuracy in predicting the diagnosis of PA (AUC=0.908). Among normokalemic patients, the ARR value that maximized the diagnostic accuracy, as identified by the Youden index, was equal to 41.8 (ng/dL)/(ng/mL/h), and was characterized by a sensitivity and a specificity of 100% and 67%, respectively (AUC=0.882); an ARR > 179.6 (ng/dL)/(ng/mL/h) provided a 100% specificity for the diagnosis of PA, but was associated with a very low sensitivity of 20%. Among hypokalemic patients, the ARR value that maximized the diagnostic accuracy, as identified by the Youden index, was equal to 49.2 (ng/dL)/(ng/mL/h), and was characterized by a sensitivity and a specificity of 100% and 83%, respectively (AUC=0.941); an ARR > 104.0 (ng/dL)/(ng/mL/h) provided a 100% specificity for the diagnosis of PA, with a sensitivity of 64%.

**Conclusions:**

Among normokalemic patients, there was a wide overlap in ARR values between those with PA and those with essential RH; the possibility to skip a confirmatory test should thus be considered with caution in this setting. A better discriminating ability could be seen in the presence of hypokalemia; in this case, ARR alone may be sufficient to skip confirmatory tests in a suitable percentage of patients.

## Introduction

Primary aldosteronism (PA) represents the most frequent cause of secondary hypertension (1, 2). Its prevalence increases with the severity of hypertension, reaching over 20% among patients with resistant hypertension (RH) (3, 4). From a clinical point of view, this condition is associated with an increased risk of cardiovascular events and target organ damage that is independent from the degree of blood pressure (BP) elevation; in fact, patients with PA display a higher risk of coronary artery disease, stroke, atrial fibrillation and heart failure compared to matched essential hypertensives with similar BP levels (3, 5–7).

The diagnosis of PA is a multi-step process, comprising screening tests, confirmatory tests, and subtype differentiation (1, 8). The measurement of the aldosterone-to-renin ratio (ARR) is the most reliable method which is currently available for screening for PA (1, 8, 9). Patients with a positive screening test are recommended to undergo confirmatory testing, either by saline infusion test, oral salt loading test, fludrocortisone suppression test, or captopril challenge test (1, 8, 10). Finally, patients in which a diagnosis of PA is confirmed should proceed to subtype testing by adrenal computed tomography (CT) and adrenal vein sampling (AVS), in order to identify any adrenal masses and to differentiate between unilateral and bilateral forms (1, 8, 11–13).

This multi-step process, however, increases the time and complexity of the diagnostic work-up, thus contributing to the underdiagnosis of PA (14). This issue is of particular relevance in patients with RH; in fact, any biochemical assessment for the diagnosis and subtype differentiation of PA should be performed after discontinuation of interfering medications (1, 8), with possible difficulties in maintaining an adequate BP control in patients with a more severe hypertensive phenotype. A finer tailoring of the diagnostic process would thus be particularly helpful to reduce the time required for diagnostic work-up in this cohort.

The actual need to go through all the steps has been questioned by some authors (15); in particular, the systematic use of confirmatory tests has been criticized, and various strategies have been proposed to skip this step (15, 16). Patients with RH, however, are characterized by a dysregulated renin-angiotensin-aldosterone system (RAAS), even in the absence of PA (17, 18). Therefore, in these patients, the generalizability of the cut-offs and flow-charts derived from unselected cohorts of non-resistant hypertensive patients is unclear.

Aim of this study was to evaluate the predictive performance of the ARR in the diagnosis of PA in a prospective cohort of patients with true RH. More specifically, we set out to determine whether specific ARR cut-offs could allow the unambiguous identification of patients with PA, without the need of a confirmatory test.

## Methods

### Patient selection and data collection

We enrolled all consecutive patients referred to our Center (Hypertension Unit, Division of Endocrinology, Diabetes and Metabolism, University of Turin) and diagnosed with true RH between September 2011 and April 2022. The following exclusion criteria were adopted: age < 18 years or > 80 years, chronic diseases with major organ involvement, chronic systemic glucocorticoid therapy, oral contraceptives, alcohol abuse, pseudo-resistant hypertension, or hypertension due to other secondary causes. The remaining patients underwent full biochemical assessment for PA, which encompassed – in all of them – both the evaluation of a basal ARR and the execution of a saline infusion test (SIT).

Overall, the following data were collected for each patient: age, sex, duration of hypertension, body mass index (BMI), systolic BP, diastolic BP, number of anti-hypertensive drugs, fasting glucose, lipid profile, creatinine, estimated glomerular filtration rate (eGFR), serum sodium, serum potassium, plasma aldosterone concentration (PAC), plasma renin activity (PRA) and PAC after SIT. Measurements of PAC and PRA were performed after the replacement of any interfering drug, according to Endocrine Society (ES) guidelines (1). Furthermore, before hormonal testing, patients were advised to maintain a normal sodium intake, and hypokalemia was corrected by oral supplementation whenever needed. PA was diagnosed when the following conditions were met at the same time: baseline PAC ≥ 15 ng/dL, ARR ≥ 40 (ng/dL)/(ng/mL/h), and PAC after SIT ≥ 10 ng/dL. To avoid a factitious inflation of the ARR value when PRA values were <0.20 ng/mL/h, a minimum PRA value of 0.20 ng/mL/h was adopted for ARR calculation.

In patients diagnosed with PA, adrenal CT and AVS were offered for subtype differentiation. All patients demonstrating a lateralization of aldosterone secretion (lateralization index > 4) underwent adrenalectomy; surgical outcomes were assessed according to the PASO criteria (19). All other patients were treated with mineralocorticoid receptor antagonists (MRA).

The study was approved by the local Ethics Committee (n. 0029505) and was in accordance with the principles of the Declaration of Helsinki. Written informed consent was obtained from all included patients.

### Blood pressure measurements

Office BP values were collected according to current guidelines (20). All patients underwent to 24-hour ambulatory BP monitoring (ABPM), using an automated, noninvasive and oscillometric device (TM-2430; Intermed S.r.l., Milan, Italy); recordings were made every 15 minutes for the daytime and every 20 minutes for the night-time. The adequacy of BP control was assessed according to current guidelines (20).

### Analytical methods

Plasma aldosterone levels (ng/dL) were measured by RIA (ACTIVE^®^ Aldosterone RIA kit, Beckman Coulter Inc., Brea, CA, USA); the sensitivity of the assay was 0.764 ng/dL; the intra- and inter-assay coefficients of variation (CV) were ≤ 4.5% and ≤ 9.8%, respectively. PRA (ng/mL/h) was assessed by radioimmunoassay (Angiotensin I RIA kit, Beckman Coulter Inc., Brea, CA, USA); the sensitivity of the assay was 0.20 ng/mL/h; the intra- and inter-assay CV were ≤ 11.3% and ≤ 20.9%, respectively. Fasting glucose, lipid profile, creatinine as well as serum sodium and potassium were assayed by clinical chemistry analyser DxC 700 AU (Beckman Coulter Inc., Brea, CA, USA).

### Statistical analysis

Normally distributed variables were summarized as mean ± standard deviation (SD); non-normally distributed variables were summarized as median [interquartile range (IQR)]; categorical variables were summarized as percent values. Kolmogorov-Smirnov test was used to assess normality. Between-group differences were evaluated by Student t-test or Mann-Whitney U test for continuous variables and by chi-squared test or Fisher’s exact test for categorical variables, as appropriate.

The accuracy of biochemical predictors in the diagnosis of PA was evaluated by the area under curve (AUC) at receiver operating characteristic (ROC) analysis. The Youden index was used for reporting the optimal ARR cut-off for PA diagnosis when equal weight is given to sensitivity and specificity. In addition, given the aim to assess whether specific ARR cut-offs could allow the unambiguous identification of patients with PA, without the need of a confirmatory test, the thresholds associated with a 100% specificity were also retrieved.

A cut-off of 0.05 was adopted for the definition of statistical significance. Statistical analysis was performed using STATA 17 (StataCorp, College Station, Texas, USA).

## Results

### General characteristics of the study population

**Figure 1:**
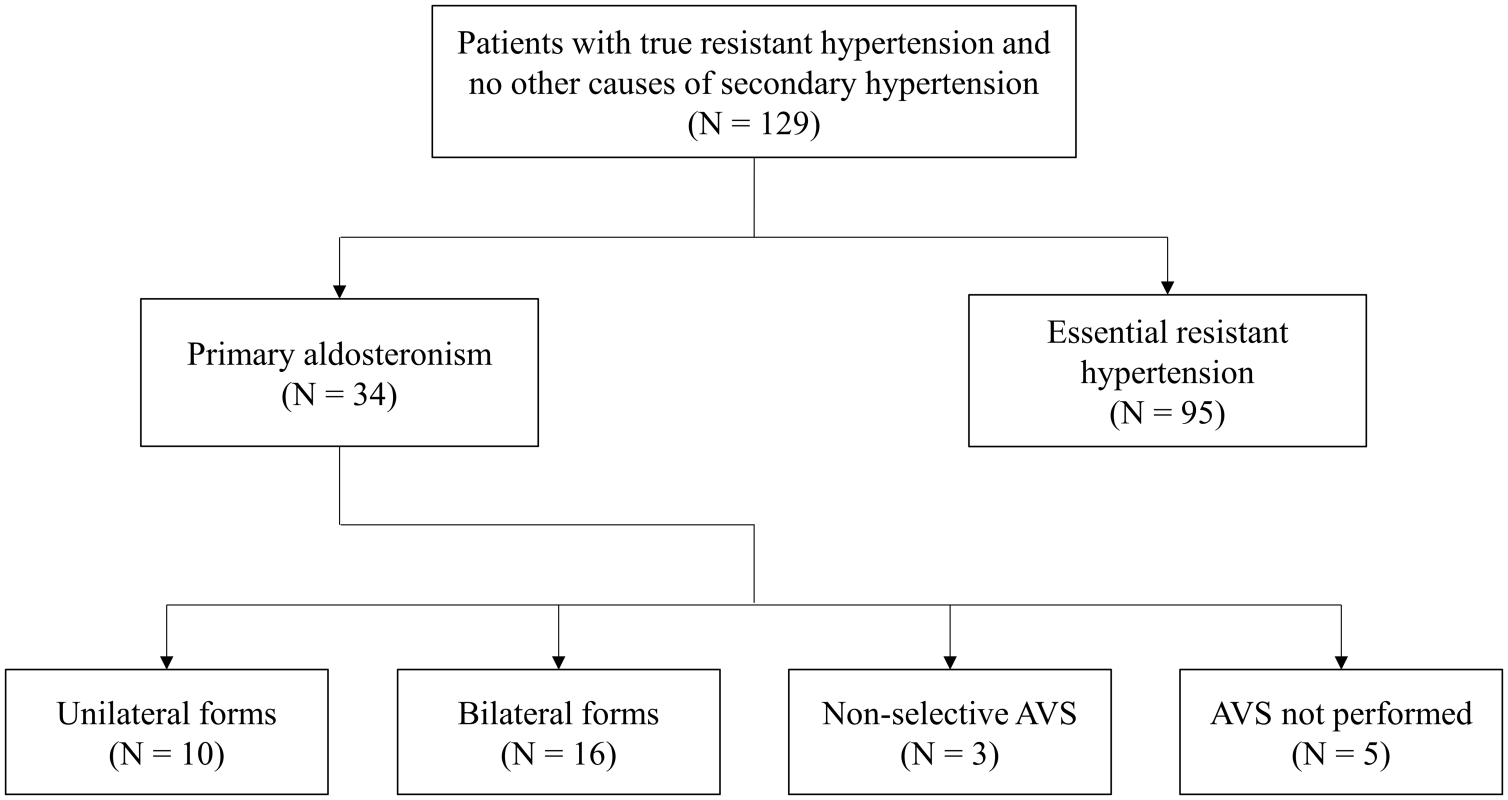
Study flow-chart. AVS, adrenal vein sampling; N, number.

After the application of all inclusion and exclusion criteria, a total of 129 patients were finally enrolled. Among these, 34 patients (26.4%) were diagnosed with PA. Subtype differentiation by AVS revealed 10 cases of unilateral PA and 16 cases of bilateral PA; in 3 cases, AVS was non-diagnostic; in other 5 cases, AVS was not performed due to patient’s choice (**Figure 1**). All cases with unilateral PA underwent adrenalectomy, while the others were referred to medical treatment with MRA.

**Table 1** reports the comparison between patients diagnosed with PA and those diagnosed with essential resistant hypertension (eRH). Patients with PA tended to be younger (54.9 ± 11.8 vs 59.3 ± 11.0 years, p=0.051), were more frequently males (74.9% vs 47.4%, p=0.001), had a higher 24-hour diastolic BP (89.1 [84.5-95.3] vs 80.6 [74.0-87.2] mmHg, p<0.001), and lower potassium levels (3.6 ± 0.5 vs 4.0 ± 0.5 mmol/L, p<0.001). Moreover, they displayed lower PRA (0.22 [0.20-0.50] vs 0.76 [0.20-2.09] ng/mL/h, p<0.001), higher PAC (36.3 [24.8-47.5] vs 19.4 [12.0-33.1] ng/dL, p<0.001), higher ARR (92.6 [68.5-199.4] vs 30.0 [13.2-52.0] (ng/dL)/(ng/mL/h), p<0.001), and higher PAC after SIT (16.9 [12.5-22.4] vs 6.4 [3.6-9.8] ng/dL, p<0.001). No difference could be found in any of the other evaluated parameters (**Table 1**).

**Table 1:** Comparison of the main clinical and biochemical characteristics between patients with PA and patients with eRH.

Variables/parameters	PA (N=34)	eRH (N=95)	p-value
Age (years)	54.9 ± 11.8	59.3 ± 11.0	0.051
Male sex (%)	79.4	47.4	0.001
Smoking habit (%)	35.3	21.1	0.100
Duration of hypertension (years)	15.1 ± 8.8	15.2 ± 10.1	0.970
24h Systolic BP at ABPM (mmHg)	149.5 [143.1-152.5]	143.9 [131.8-153.6]	0.145
24h Diastolic BP at ABPM (mmHg)	89.1 [84.5-95.3]	80.6 [74.0-87.2]	<0.001
N of anti-hypertensive medications	4 [4-5]	4 [4-5]	0.817
BMI (kg/m^2^)	29.3 ± 4.2	30.5 ± 5.9	0.276
Glucose (mg/dL)	98.2 ± 22.7	103.9 ± 28.8	0.306
Total cholesterol (mg/dL)	196.4 ± 42.8	198.7 ± 40.8	0.788
Triglycerides (mg/dL)	110.8 ± 56.5	130.4 ± 85.0	0.215
HDL cholesterol (mg/dL)	53.8 ± 11.6	52.8 ± 14.8	0.724
LDL cholesterol (mg/dL)	120.5 ± 39.2	120.3 ± 36.7	0.982
Creatinine (mg/dL)	0.89 ± 0.21	0.85 ± 0.21	0.265
eGFR (CKD-EPI, mL/min/1.73m^2^)	89.9 ± 17.4	87.1 ± 16.8	0.411
Serum sodium (mmol/L)	141.7 ± 1.6	141.5 ± 2.5	0.698
Serum potassium (mmol/L)	3.6 ± 0.5	4.0 ± 0.5	<0.001
Hypokalemia (%)	41.2	8.4	<0.001
PRA (ng/mL/h)	0.22 [0.20-0.50]	0.76 [0.20-2.09]	<0.001
PAC (ng/dL)	36.3 [24.8-47.5]	19.4 [12.0-33.1]	<0.001
ARR ((ng/dL)/(ng/mL/h))	92.6 [68.5-199.4]	30.0 [13.2-52.0]	<0.001
PAC after SIT (ng/dL)	16.9 [12.5-22.4]	6.4 [3.6-9.8]	<0.001

ARR, aldosterone-to-renin ratio; BMI, body mass index; BP, blood pressure; CKD-EPI, chronic kidney disease epidemiology collaboration; eGFR, estimated glomerular filtration rate; eRH, essential resistant hypertension; HDL, high-density lipoprotein; LDL, low-density lipoprotein; N, number; PA, primary aldosteronism; PAC, plasma aldosterone concentration; PRA, plasma renin activity; SIT, saline infusion test.

### Predictive performance of ARR for the diagnosis of PA

**Figure 2:**
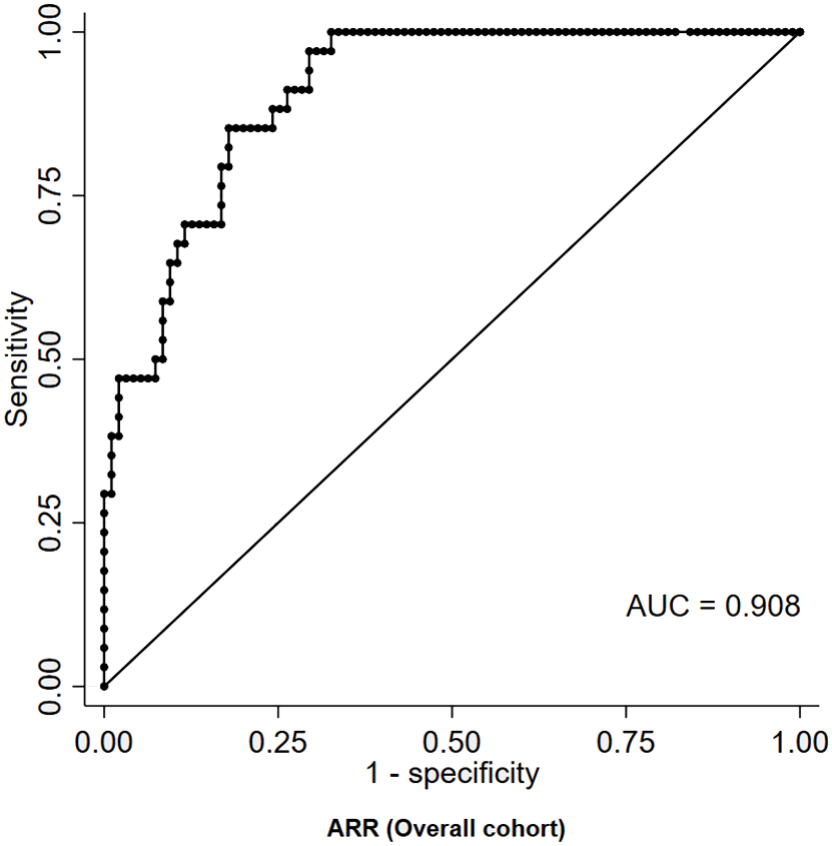
ROC curve of ARR for the diagnosis of PA in patients with RH. ARR, aldosterone-to-renin ratio; AUC, area under curve; PA, primary aldosteronism; RH, resistant hypertension; ROC, receiver-operating characteristic.

Among basal measurements, the parameter that provided the best diagnostic performance was represented by ARR, which displayed a moderate-to-high accuracy in distinguishing patients with PA from those with eRH (AUC=0.908, **Figure 2**). The diagnostic accuracy of all other basal measurements was significantly inferior to that of ARR (AUC for PAC: 0.766; AUC for PRA: 0.725; AUC for serum potassium: 0.729; p<0.05 for inferiority to ARR in all comparisons; **Supplementary Figure 1**). The ARR value that maximized the diagnostic accuracy, as identified by the Youden index, was equal to 43.6 (ng/dL)/(ng/mL/h); this cut-off was characterized by a sensitivity and a specificity of 97% and 71%, respectively. The ARR cut-off that ensured perfect specificity (100%) was equal to 179.6 (ng/dL)/(ng/mL/h); however, this was associated to a marked reduction in sensitivity, which dropped to 29% (**Table 2**).

**Table 2:** ARR cut-offs associated with a 100% specificity for the diagnosis of PA in patients with RH, stratified according to the absence or presence of hypokalemia.

	Overall cohort	Normokalemic patients	Hypokalemic patients
ARR cut-off ((ng/dL)/(ng/mL/h))	> 179.6	> 179.6	> 104.0
Specificity (%)	100%	100%	100%
Sensitivity (%)	29%	20%	64%

ARR, aldosterone-to-renin ratio; PA, primary aldosteronism; RH, resistant hypertension.

At multivariable logistic regression, ARR (OR 1.03, 95%CI 1.02-1.05, p<0.001) and hypokalemia (OR 3.89, 95%CI 1.04-14.64, p=0.044) were identified as independent significant predictors of PA; PRA and PAC were excluded from the multivariable analysis due to their strong collinearity with ARR. In order to improve predictive accuracy, patients were thus stratified according to the presence/absence of hypokalemia.

**Figure 3:**
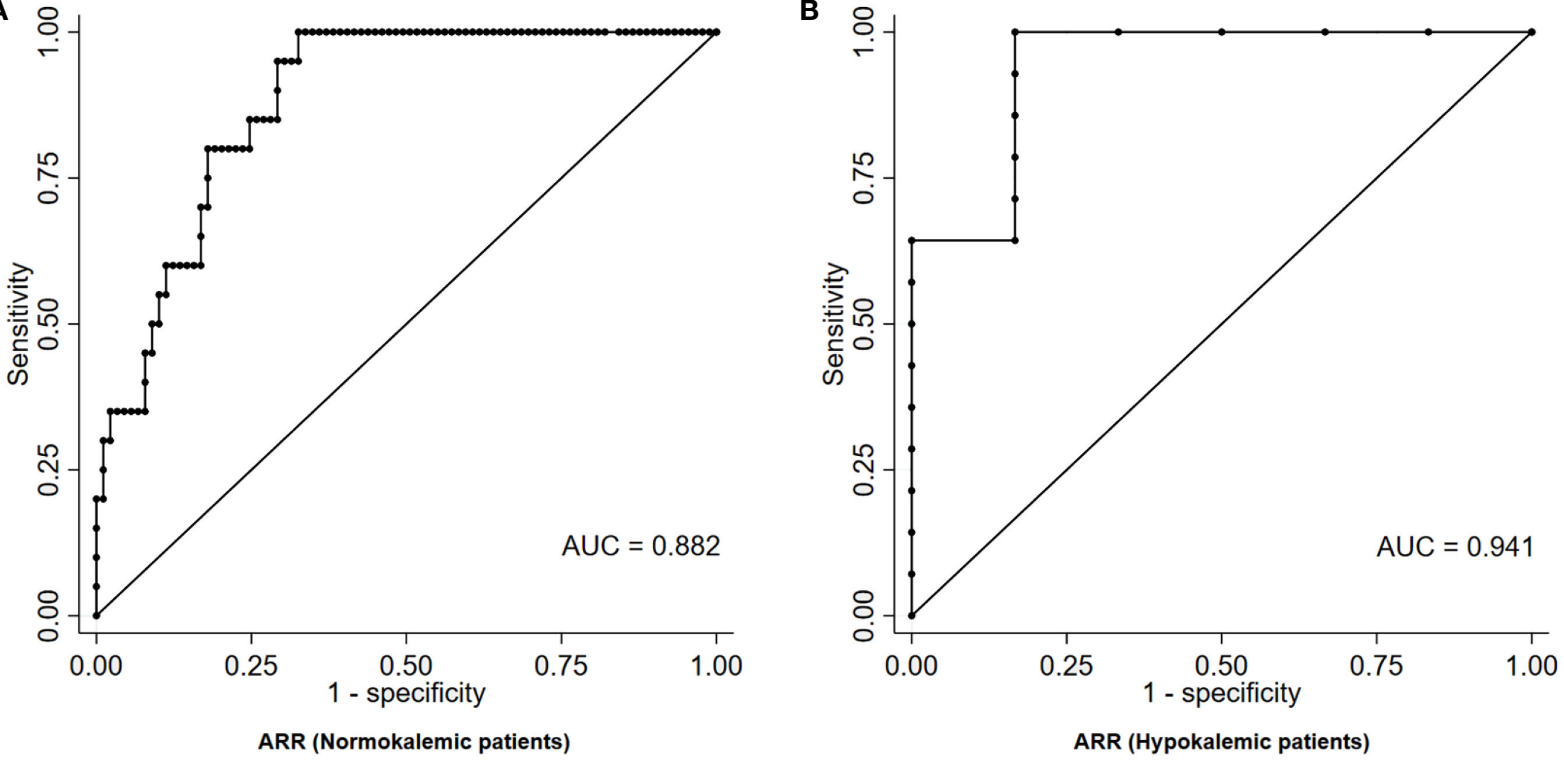
ROC curve of ARR for the diagnosis of PA in patients with RH, stratified according to the absence **(A)** or presence **(B)** of hypokalemia. ARR, aldosterone-to-renin ratio; AUC, area under curve; PA, primary aldosteronism; RH, resistant hypertension; ROC, receiver-operating characteristic.

Among normokalemic patients, the diagnostic performance of ARR was slightly lower than in the overall cohort, with an AUC of 0.882 at ROC analysis (**Figure 3A**). The ARR value that maximized the diagnostic accuracy, as identified by the Youden index, was equal to 41.8 (ng/dL)/(ng/mL/h), and was characterized by a sensitivity and a specificity of 100% and 67%, respectively. An ARR > 179.6 (ng/dL)/(ng/mL/h) provided a 100% specificity for the diagnosis of PA in normokalemic patients, but was associated with a very low sensitivity of 20% (**Table 2**). Even allowing for a less strict specificity cut-off of 95%, which corresponded to an ARR > 119.2 (ng/dL)/(ng/mL/h), the sensitivity only slightly improved to 35%.

Among hypokalemic patients, on the contrary, the diagnostic performance of ARR was higher than in the overall cohort, with an AUC of 0.941 at ROC analysis (**Figure 3B**). The ARR value that maximized the diagnostic accuracy, as identified by the Youden index, was equal to 49.2 (ng/dL)/(ng/mL/h), and was characterized by a sensitivity and a specificity of 100% and 83%, respectively. An ARR > 104.0 (ng/dL)/(ng/mL/h) provided a 100% specificity for the diagnosis of PA in hypokalemic patients, with a sensitivity of 64% (**Table 2**).

As a term of comparison, the diagnostic performance of the current criteria of ES guidelines (1), according to which confirmatory testing could be skipped in patients with spontaneous hypokalemia, undetectable renin, and PAC > 20 ng/dL, was also evaluated in our cohort. Overall, only 4 patients fulfilled them (all diagnosed with PA). Thus, despite having optimal specificity (100%), these criteria were characterized by a very low sensitivity (12% in the overall cohort, 29% when restricting the evaluation to hypokalemic patients).

## Discussion

In this study, we specifically assessed the predictive performance of ARR for the diagnosis of PA in patients with RH; in addition, we proposed specific ARR cut-offs above which a confirmatory test may be avoided, differentiated according to the presence/absence of concurrent hypokalemia.

The diagnosis of PA is a multi-step process, comprising screening tests, confirmatory tests, and subtype differentiation. The systematic use of confirmatory tests in all patients with a positive screening test, however, determines an increase in costs, time, and complexity in the management of patients with PA (21), thus probably contributing to the underdiagnosis of this condition (22). In light of this, various authors proposed ARR cut-offs and/or integrated diagnostic flow-charts to identify patients in which a confirmatory test for PA could be avoided. Nanba et al. demonstrated that, in patients evaluated for PA, the diagnosis could be confirmed in most cases with ARR ≥ 100 (ng/dL)/(ng/mL/h), PAC ≥ 25 ng/dL and suppressed renin (10). Maiolino et al. displayed that increasing ARR values were associated with an exponential increase of the likelihood of an aldosterone-producing adenoma (15). According to the ES guidelines (1) and to the European Society of Hypertension (ESH) consensus (23), confirmatory testing could be skipped in patients with spontaneous hypokalemia, undetectable renin, and PAC > 20 ng/dL. Finally, two recent studies developed two different scoring systems to skip confirmatory testing, by adopting different combinations of predictive parameters, such as age, sex, BMI, antihypertensive medications, sodium, potassium, PRA and aldosterone values, presence of diabetes, and presence of organ damage (16, 24).

Patients with RH represent a population in which a correct diagnosis of PA is particularly crucial. In fact, PA is a highly prevalent condition among patients with a RH, reaching over 20% in some studies (3, 4); moreover, both PA and RH are “high-risk phenotypes”, associated with increased cardiovascular morbidity and mortality compared to non-PA and non-RH patients (5, 17, 25, 26). The implementation of an effective and targeted therapy plays a key role in the management of these patients, not only for the improvement of BP values (19), but also for the reduction of PA-related cardiovascular risk (27, 28). Notably, a recent study by Rossi et al. clearly demonstrated that unilateral adrenalectomy resolved resistance to antihypertensive treatment in almost all patients with PA and RH who underwent surgery (29). Therefore, missing a diagnosis of PA would be particularly relevant in this context.

In patients with RH, however, the burden in terms of time and complexity of the diagnostic process of PA is even higher than in non-RH patients. In fact, the maintenance of an adequate BP control using only non-interfering medications might be difficult in case of a more severe hypertensive phenotype. Moreover, the occurrence of symptoms or side effects during confirmatory tests has been shown to be associated with both with higher BP values and with a higher number of anti-hypertensive drugs (21). The availability of strategies to simplify the diagnostic work-up for PA would be, therefore, even more relevant in RH patients.

It is unclear, however, whether the previously discussed ARR cut-offs and/or integrated diagnostic flow-charts might be applied with the same diagnostic reliability in the setting of RH. Patients with RH, in fact, are characterized by a dysregulated RAAS, with a common finding of low-renin states and relative aldosterone excess, even in the absence of a defined diagnosis of PA (17, 18). In a study by Gaddam et al., the authors showed that higher PAC and lower PRA values in patients with RH compared to those without (30). Moreover, the PATHWAY-2 study distinctly demonstrated that the add-on treatment with a MRA, even in the absence of PA, was the most effective strategy to lower blood pressure in patients with RH (31); notably, in this trial, the response to MRA treatment was directly related to aldosterone and inversely related to renin, being spironolactone particularly effective in patients’ higher aldosterone and lower renin levels (31). Overall, all these findings further support an active pathogenetic role of RAAS dysregulation in the development and maintenance of a RH phenotype.

In our study, among basal measurements, the one that provided the highest diagnostic accuracy was represented by ARR. This was coherent with previous literature data derived in unselected cohorts of resistant and non-resistant hypertensives, in which ARR was demonstrated to be superior in diagnosing PA than potassium, aldosterone (both being less sensitive), or renin (being less specific) in isolation (32–34).

Overall, the diagnostic performance of ARR in the diagnosis of PA in our cohort was moderate-to-high, meaning that ARR remains a reliable tool for the identification of PA patients also in the setting of RH. However, a perfect specificity in PA diagnosis could be achieved only with high ARR values and at the cost of a remarkable decline in sensitivity. After stratifying the analysis according serum potassium levels, the diagnostic accuracy of ARR was higher in patients with hypokalemia than in those with normokalemia. In the first subgroup, a perfect specificity could be achieved while maintaining a satisfactory sensitivity of 64%, with a ARR cut-off > 104.0 (ng/dL)/(ng/mL/h). On the other hand, in the second subgroup, the requirement of a perfect specificity corresponded to a marked reduction in sensitivity to 20%, with a ARR cut-off > 179.6 (ng/dL)/(ng/mL/h).

This difference in the diagnostic performance of ARR underlines the key role of hypokalemia as a distinctive feature of PA. In fact, with normal potassium levels, the overlap in ARR values between PA and eRH patients was wide, thus limiting the chances to avoid confirmatory testing based on ARR alone. On the other hand, the presence of hypokalemia was able to significantly strengthen ARR diagnostic accuracy, enhancing its sensitivity, and thus broadening the possibility to skip confirmatory tests.

The main strength of this study was the prospective enrollment of consecutive patients diagnosed with RH; all of them have been submitted to careful and standardized evaluations, in order to identify and exclude all patients with pseudo-resistant hypertension and/or with hypertension due to other secondary causes; moreover, a full diagnostic work-up for PA, comprising both screening and confirmatory tests, was performed in all enrolled subjects. Our study had also some limitations. First, since there is no unanimous consensus on the criteria to be adopted for the definition of PA, the criteria adopted in this paper represent only one of the possible choices among those currently suggested by international guidelines (1). Second, the generalizability of our results could be limited depending on the specific assays used for PAC and PRA measurement. Third, a referral bias might be present, due to the tertiary nature of our center. Fourth, since all enrolled patients were of caucasian ethnicity, the applicability of these results in other non-caucasian populations is unclear.

In conclusion, our results pointed out that, among normokalemic RH patients, only very high values of ARR were diagnostic for PA, and a perfect specificity could be achieved only at the expense of a significant reduction in sensitivity; this wide overlap in ARR values is likely an effect of the functional dysregulation of RAAS which frequently underlies the pathophysiology of eRH; as a consequence, the possibility to skip a confirmatory test should be considered with caution in this setting. On the other hand, the presence of hypokalemia was able to enhance ARR diagnostic performance, allowing for a better discriminating ability between PA and eRH patients; in this case, ARR values may be sufficient to avoid confirmatory tests in a suitable percentage of patients.

## Data availability statement

The raw data supporting the conclusions of this article will be made available by the authors, without undue reservation.

## Ethics statement

The studies involving human participants were reviewed and approved by Comitato Etico Interaziendale A.O.U. Città della Salute e della Scienza di Torino. The patients/participants provided their written informed consent to participate in this study.

## Author contributions

FB contributed to work conceptualization, data collection, data analysis and manuscript writing. CL, MB, SA and MP contributed to data collection. FP and GB contributed to manuscript review and editing. GM and EG supervised the manuscript drafting. MM and MP-C contributed to work conceptualization, data collection, data analysis, manuscript writing and final draft supervision. All authors contributed to the article and approved the submitted version.
