# Supplementary material for: Predictive performance of aldosterone-to-renin ratio in the diagnosis of primary aldosteronism in patients with resistant hypertension
Source: Front Endocrinol (Lausanne). 2023 May 8;14:1145186. doi: 10.3389/fendo.2023.1145186 (PMC10200868; doi:10.3389/fendo.2023.1145186)

Supplementary Material

Predictive performance of aldosterone-to-renin ratio in the diagnosis of primary aldosteronism in patients with resistant hypertension

Fabio Bioletto[1*], Chiara Lopez[1], Martina Bollati[1], Stefano Arata[1], Matteo Procopio[1], Federico Ponzetto[1], Guglielmo Beccuti[1], Giulio Mengozzi[2], Ezio Ghigo[1], Mauro Maccario[1], Mirko Parasiliti-Caprino[1]

[1] Division of Endocrinology, Diabetes and Metabolism; Department of Medical Sciences; University of Turin; Turin, Italy

[2] Clinical Biochemistry Laboratory; City of Health and Science University Hospital; Turin, Italy

*** Corresponding author:**

Fabio Bioletto, MD

Endocrinology, Diabetes and Metabolism

Department of Medical Sciences, University of Turin

Corso Dogliotti 14, Turin 10126, Italy

+39 0116335544

fabio.bioletto@unito.it

ORCID iD 0000-0001-7550-7023

Supplementary Figure 1. ROC curves of PAC (A), PRA (B) and serum potassium (C) for the diagnosis of PA in patients with RH. Abbreviations: ARR, aldosterone-to-renin ratio; AUC, area under curve; PA, primary aldosteronism; PAC, plasma aldosterone concentration; PRA, plasma renin activity; RH, resistant hypertension; ROC, receiver-operating characteristic.

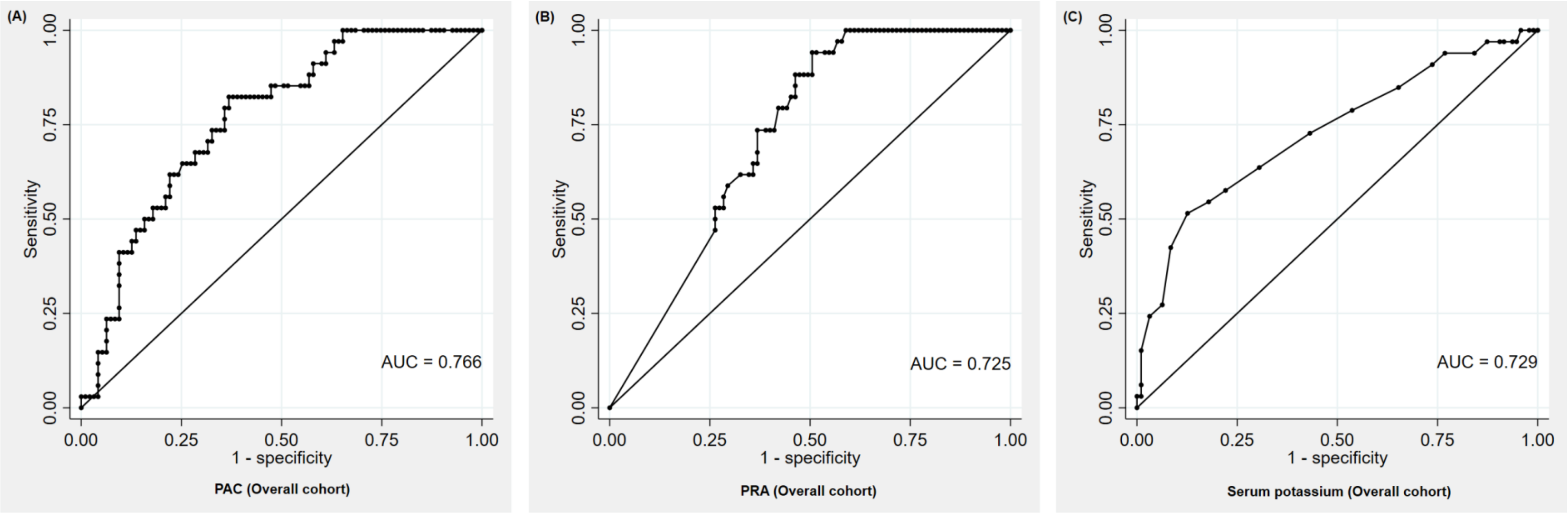

Supplement: Supplementary file 1 [file DataSheet_1.pdf]
